# Impact of the COVID-19 pandemic on the clinical management trends for acute appendicitis among the under-25s: a retrospective study

**DOI:** 10.1136/archdischild-2023-326313

**Published:** 2024-02-07

**Authors:** Puji Faitna, Rachel Harwood, Simon E Kenny, Russell M Viner, Paul P Aylin, Dougal S Hargreaves, Alex Bottle

**Affiliations:** 1 Department of Primary Care and Public Health, School of Public Health, Imperial College London, London, UK; 2 Department of Paediatric Surgery, Alder Hey Children's Hospital, Liverpool, UK; 3 National Clinical Director for Children and Young People, NHS England and NHS Improvement, London, UK; 4 Institute of Systems, Molecular and Integrative Biology, University of Liverpool, Liverpool, UK; 5 Population, Policy and Practice Research Programme, UCL Institute of Child Health, London, UK; 6 Mohn Centre for Children's Health and Wellbeing, Imperial College London, London, UK

**Keywords:** Covid-19, Emergency Care, Epidemiology, Paediatric Emergency Medicine, Statistics

## Abstract

**Objective:**

To describe the COVID-19 pandemic’s impact on acute appendicitis management on children and young people (CYP).

**Design:**

Retrospective cohort study.

**Setting:**

All English National Health Service hospitals.

**Patients:**

Acute appendicitis admissions (all, simple, complex) by CYP (under-5s, 5–9s, 10–24s).

**Exposure:**

Study pandemic period: February 2020–March 2021. Comparator pre-pandemic period: February 2015–January 2020.

**Main outcome measures:**

Monthly appendicectomy and laparoscopic appendicectomy rate trends and absolute differences between pandemic month and the pre-pandemic average. Proportions of appendicitis admissions comprising complex appendicitis by hospital with or without specialist paediatric centres were compared.

**Results:**

101 462 acute appendicitis admissions were analysed. Appendicectomy rates fell most in April 2020 for the 5–9s (−18.4% (95% CI −26.8% to −10.0%)) and 10–24s (−28.4% (−38.9% to −18.0%)), driven by reductions in appendicectomies for simple appendicitis. This was equivalent to −54 procedures (−68.4 to −39.6) and −512 (−555.9 to −467.3) for the 5–9s and 10–24s, respectively. Laparoscopic appendicectomies fell in April 2020 for the 5–9s (−15.5% (−23.2% to −7.8%)) and 10–24s (−44.8% (−57.9% to −31.6%) across all types, which was equivalent to −43 (−56.1 to 30.3) and −643 (−692.5 to −593.1) procedures for the 5–9s and 10–24s, respectively. A larger proportion of complex appendicitis admissions were treated within trusts with specialist paediatric centres during the pandemic.

**Conclusions:**

For CYP across English hospitals, a sharp recovery followed a steep reduction in appendicectomy rates in April 2020, due to concerns with COVID-19 transmission. This builds on smaller-sized studies reporting the immediate short-term impacts.

**WHAT IS ALREADY KNOWN ON THIS TOPIC**

- COVID-19 studies on how acute appendicitis was clinically managed have often focused on smaller numbers of hospitals, adult populations and fewer pandemic waves.
- Most published studies in this area have focused on the first few pandemic weeks and compared this only with the previous year.

**WHAT THIS STUDY ADDS**

- This is the first study to capture all acute appendicitis hospital admissions among the under-25s in England from February 2015 to March 2021.
- There were large changes in appendicectomy and laparoscopic appendicectomy rates for simple appendicitis during the pandemic but relatively stable procedure rates for complex appendicitis.
- Pre-pandemic trends towards centralisation of care and reduced variation in surgery rates for children with complex appendicitis in non-specialist centres continued during the pandemic.

**HOW THIS STUDY MIGHT AFFECT RESEARCH, PRACTICE OR POLICY**

- These findings reinforce the importance of early recognition and referral of appendicitis by paediatricians, general practitioners and all acute child health professionals.
- The COVID-19 pandemic may have strengthened previous efforts to address variations in complex appendicitis by hospital, but future research could explore what impact the pandemic had on variations in the rate of negative appendicectomy or laparoscopic appendicectomy.
- These findings can be used to better understand how COVID-19 impacted the management of appendicitis, providing a national picture of how hospitals dealt with acute appendicitis admissions in the first 14 months of the pandemic.

## Introduction

The COVID-19 pandemic disrupted hospital services worldwide,^1 2^ and early studies reported significant declines in surgeries, in part to ensure capacity for surges in COVID-19 admissions and to reduce possible exposure risk through aerosol-generating procedures (AGPs) within hospitals.^3 4^ While SARS-CoV-2 produces a mild, self-limiting viral illness in most children and young people (CYP),^5–7^ the indirect impacts of the pandemic on CYP’s health appear to be significant.^8^

Appendicitis, one of the most common surgical emergencies affecting CYP, is a useful condition to better understand the pandemic’s impacts on CYP. A national initiative^9^ has highlighted disparities in care for CYP with appendicitis and has recommended improving access to laparoscopic surgery, in contrast to open surgery, in younger children and standardising postoperative care as key approaches to addressing these disparities in care. Paediatricians and the multidisciplinary health teams play a pivotal role in supporting improvements in patient care for CYP with appendicitis^10 11^ as they are experienced in dealing with unexplained symptoms in CYP^10^ and are frequently the first point of contact.^12^

Smaller-scale studies have reported changes in the care of CYP with appendicitis during the pandemic,^13–15^ but to fully appreciate this impact, measuring changes over a longer pandemic period and understanding pre-pandemic trends beyond the previous year are needed. Key pre-pandemic changes in care include the increased provision of laparoscopic surgery and non-operative management in simple appendicitis,^16–19^ which likely changed during the pandemic when early guidance cautioned against using laparoscopy as it is an AGP.^20 21^ In the UK, acute appendicitis is generally treated by general or paediatric surgeons within specialist paediatric centres,^22^ and there is ongoing debate about where paediatric surgeries are conducted and how this may impact disparity in outcomes.^21 23^

Acute appendicitis is a high-volume time-sensitive paediatric surgical condition, where presentation delays may result in higher complex appendicitis rates. The pandemic’s impact on acute surgical activity in the UK needs to be better understood in the context of pre-pandemic trends and beyond the first few pandemic weeks for CYP.

### Primary aims

To describe the monthly impact COVID-19 had on appendicectomy and laparoscopic appendicectomy rates and if any changes persisted.

### Secondary aim

To explore changes to the proportion of complex appendicitis admissions by hospital between study periods.

## Methods

### Data sources and cohort definition

England’s Hospital Episode Statistics (HES) admitted patient care data were analysed and individuals with at least one hospital spell were extracted. Further information on HES is available elsewhere.^24^ At the time of analysis, data were available up to March 2021. Transfers between consultants and hospitals were linked into ‘superspells’ and referred to as admissions.

Admissions by patients under-25 were extracted. Acute appendicitis was defined using codes provided elsewhere^25^ (online supplemental tables A–C). The WHO age categories were used: 0–4 (under-5s), 5–9 (5–9s) and 10–24 years (10–24s). Although local protocols may vary, these age groups broadly reflect National Health Service pathways whereby most children under-5 years with appendicitis would be transferred to a specialist centre, while most aged 10 years or over would be treated in a District General Hospital.

10.1136/archdischild-2023-326313.supp1 Supplementary data

Acute appendicitis was analysed into three subgroups: (1) all (2) simple and (3) complex (online supplemental table A). K35 was stratified into simple and complex appendicitis. Clinical coders use histopathology to code appendicitis. Admissions with diagnosis codes for simple and complex appendicitis were classified as complex, as we assumed that these admissions reflected admissions that started as simple appendicitis and, within the course of the admission, developed into complex appendicitis.

### Exposure and outcomes

The pre-pandemic period was 1 February 2015–31 January 2020. The pandemic period was 1 February 2020–31 March 2021, covering three national lockdowns approximately defined as March–June 2020, November 2020 and January–March 2021.^25^ The pandemic period included February 2020, as the first cases of COVID-19 diagnosed in England occurred in late January 2020.^26^

#### Primary outcomes

Monthly appendicectomy and laparoscopic appendicectomy rate trends, differences in rates and counts between each pandemic month and the average of the same month for 5 pre-pandemic years, and if changes persisted beyond the first pandemic wave. When we refer to absolute differences, we refer to the difference in admission counts. The numerator and denominator for the monthly appendicectomy rate were the number of acute appendicitis admissions treated with appendicectomy divided by the number of acute appendicitis admissions, respectively. For monthly laparoscopic appendicectomy rates, the numerator and denominator were defined as the number of acute appendicitis admissions treated with laparoscopic appendicectomy and the number of acute appendicitis admissions treated with appendicectomy, respectively. Further details on how appendicectomy and laparoscopic appendicectomy were coded can be found in the online supplemental table B.

#### Secondary outcomes

Proportion of complex appendicitis admissions by hospital trust (a trust may comprise more than one hospital site) between pandemic periods.

### Statistical analyses

Pre-pandemic trends were plotted. Graphically, the pre-pandemic monthly mean over the five5 pre-pandemic years links to the pandemic monthly count or rate. To avoid seasonal biases, the difference between the observed and expected monthly figures was reported based on the pre-pandemic mean.

Funnel plots compared crude complex appendicitis admissions by hospital between pandemic periods. Funnel plots were used to identify which hospitals had significantly higher or lower proportions of crude complex appendicitis relative to the other trusts in the plot. To ensure hospital sample sizes were comparable between pandemic periods and not unfairly penalise larger hospitals that may treat a higher proportion of complex appendicitis admissions, the 14 pandemic months were compared with the preceding 14 pre-pandemic months. As control limits for the funnel plot are not robust when admission counts are low, hospitals that had fewer than 50 appendicitis admissions in either the pre-pandemic or pandemic period were excluded. Trusts that provided specialist paediatric services were highlighted.^27^

An interrupted time series (ITS) analysis was performed to establish if rate changes in the first pandemic wave persisted beyond it. ITS enables the comparison of the pre-pandemic trend with the trend after the first pandemic wave, which is important as considerable pre-pandemic trends occurred before the pandemic. By accounting for pre-pandemic trends, we are able to statistically test for any changes in these trends that occur after the first pandemic wave. ITS modelled patient-level data for better granularity; the interaction variables were coded as an ascending continuous month variable and a dichotomous pandemic variable, where ‘1’ was the pandemic period.

Differences in appendicectomy and laparoscopic appendicectomy rates between periods and the last quarter of the study were tested using Pearson’s Χ^2^ test. Where expected counts were less than five, Fisher’s exact test was used.

All analyses used SAS software V.9.4, and the statistically significant threshold was 0.05.

### Sensitivity analysis

The appendicitis definition for the sensitivity analysis is in online supplemental tables A–E. The main and sensitivity definitions were compared.

## Results

Table 1 describes the patient characteristics. 625 (0.6%) admissions were coded as both simple and complex within the same admission.

**Table 1:** Patient characteristics

Feature	Value	Acute appendicitis	
		Pre-pandemic N=83 590 (%)	Pandemic N=17 872 (%)
Appendicitis classification	Simple appendicitis (K35.8)	70 756 (84.6)	9882 (55.3)
	Complex appendicitis (K35.2, K53.3)	9882 (11.8)	5716 (32.0)
	All (including unspecified, K37)	83 590 (100.0)	17 872 (100.0)
Age	Mean (SD)	15.4 (5.5)	15.5 (5.5)
	0–4	1624 (1.9)	368 (2.1)
	5–9	12 679 (15.2)	2853 (16.0)
	10–24	69 287 (82.9)	14 651 (82.0)
Gender	Male	48 156 (57.6)	10 575 (59.2)
	Female	35 434 (42.4)	7297 (40.8)
Deprivation quintile	1 (least deprived)	16 848 (20.2)	3825 (21.4)
	2	15 803 (18.9)	3426 (19.2)
	3	15 202 (18.2)	3304 (18.5)
	4	15 983 (19.1)	3343 (18.7)
	5 (most deprived)	18 823 (22.5)	3878 (21.7)
	6 (unknown)	931 (1.1)	96 (0.5)
Ethnic group	Black or Black British	1755 (2.1)	366 (2.1)
	Asian or Asian British	5780 (6.9)	1329 (7.4)
	White	61 393 (73.5)	12 857 (71.9)
	Other (including mixed)	4635 (5.5)	1071 (6.0)
	Unknown	10 027 (12.0)	2249 (12.6)
Admission source	Home	78 079 (93.4)	16 655 (93.2)
	Transfers from acute hospital	478 (0.6)	122 (0.7)
	Transfers from other hospital	4417 (5.3)	981 (5.5)
	Other/unknown	616 (0.7)	114 (0.6)
Emergency admissions in previous 12 months	0	73 488 (87.9)	15 552 (87.0)
	1	7866 (9.4)	1837 (10.3)
	2	1457 (1.7)	312 (1.8)
	3+	779 (0.9)	171 (1.0)

### Appendicectomy

**Figure 1:**
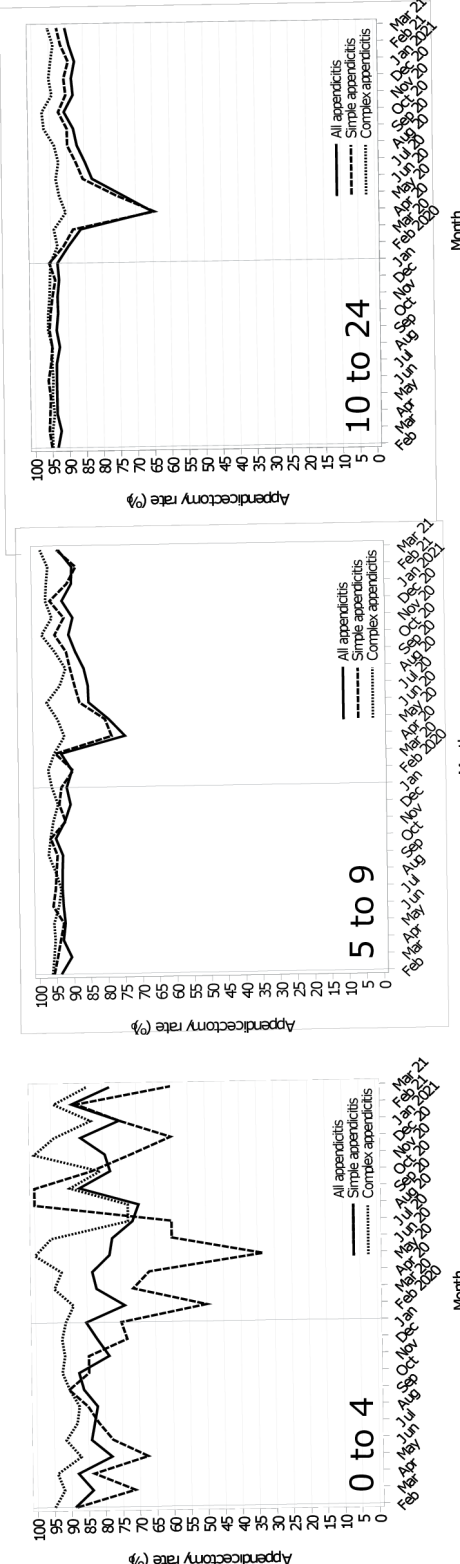
Monthly appendicectomy rate trends during the pandemic and the mean monthly rates for the previous 5 years, stratified by appendicitis type and age group.

The proportion of appendicitis admissions treated surgically via an appendicectomy was slowly declining over the last five years before the pandemic (online supplemental figure 1). During the pandemic, rates for the 5–24s reduced significantly (figure 1). The largest reduction was among the 10–24s in April (−28.4% (−38.9 to −18.0)) and May 2020 (−19.7% (−28.5 to −11.0)), driven by fewer appendicectomies for simple appendicitis (online supplemental tables 1–4). The absolute reductions were −54 appendicectomies (−68 to −40) for the 5–9s and −512 appendicectomies (−556 to −467)) for the 10–24s in April. A similar reduction was observed in May (5–9s: −39 (−52 to -27); 10–24s: −424 (−465 to −384)). A small number of under-5s were diagnosed with appendicitis (online supplemental tables 5 and 6), but a reduction in appendicectomy rates for simple appendicitis was shown.

ITS analysis demonstrated that the odds of surgical treatment (appendicectomy) after the first pandemic wave, compared with the pre-pandemic period, dropped significantly for all admissions (under-5s: 0.39 (0.19 to 0.80), p=0.0102; 5–9s: 0.33 (0.24 to 0.45), p<0.0001; 10–24s: 0.31 (0.27 to 0.36), p<0.0001) (table 2). Fewer surgeries for complex admissions drove this for the under-5s (OR 0.22 (0.08 to 0.59), p=0.0028) and for simple and complex admissions for the older age groups. For each month after the first wave, treatment with appendicectomy for all appendicitis was statistically significant, but with small effect sizes (all: under-5s: OR 0.99 (0.98 to 1.00), p=0.025; 5–9s and 10–24s: 0.99 (0.99 to 0.99), p<0.0001). The appendicectomy rate slope recovered after the first wave and remained unchanged for the under-5s (OR: 1.08 (0.95 to 1.23), p=0.2249) but was significantly steeper for the older CYP (5–9 s: OR 1.10 (1.04 to 1.16), p=0.001; 10–24s: 1.05 (1.03 to 1.08), p<0.0001).

**Table 2:** Summary of the interrupted time series analysis for appendicectomy and laparoscopic appendicectomy rate of the 5 pre-pandemic years compared with the rates after the first pandemic wave

Appendicectomy						
	All		Simple		Complex	
	OR (95% CI)	P value	OR (95% CI)	P value	OR (95% CI)	P value
**Under-5s**						
Month counter (A)	0.99 (0.98 to 1.00)	0.025*	0.99 (0.97 to 1.00)	0.0674	0.99 (0.98 to 1.01)	0.312
COVID-19 flag (B)	0.39 (0.19 to 0.80)	0.0102*	1.00 (0.19 to 5.18)	0.9979	0.22 (0.08 to 0.59)	0.0028*
A×B	1.08 (0.95 to 1.23)	0.2249	0.92 (0.70 to 1.19)	0.5119	1.18 (0.98 to 1.43)	0.0783
**5–9s**						
Month counter (A)	0.99 (0.99 to 0.99)	<0.0001*	1.00 (0.99 to 1.00)	0.2594	0.99 (0.98 to 1.00)	0.0229*
COVID-19 flag (B)	0.33 (0.24 to 0.45)	<0.0001*	0.50 (0.29 to 0.85)	0.0106*	0.37 (0.18 to 0.75)	0.006*
A×B	1.10 (1.04 to 1.16)	0.001*	1.04 (0.95 to 1.14)	0.3956	1.20 (1.04 to 1.39)	0.0121*
**10–24s**						
Month counter (A)	0.99 (0.99 to 0.99)	<0.0001*	0.99 (0.98 to 0.99)	<0.0001*	0.99 (0.99 to 1.00)	0.0013*
COVID-19 flag (B)	0.31 (0.27 to 0.36)	<0.0001*	0.25 (0.20 to 0.31)	<0.0001*	0.68 (0.46 to 0.99)	0.0437*
A×B	1.05 (1.03 to 1.08)	<0.0001*	1.06 (1.02 to 1.10)	0.001*	1.02 (0.96 to 1.09)	0.5334

Laparoscopic appendicectomy						
Under-5s	OR (95% CI)	P value	OR (95% CI)	P value	OR (95% CI)	P value
Month counter (A)	1.02 (1.02 to 1.03)	<0.0001*	1.03 (1.02 to 1.04)	<0.0001*	1.02 (1.02 to 1.03)	<0.0001*
COVID-19 flag (B)	2.53 (1.23 to 5.23)	0.0118*	3.96 (0.83 to 18.89)	0.0847	2.16 (0.92 to 5.02)	0.0753
A×B	1.11 (0.97 to 1.28)	0.1272	1.03 (0.77 to 1.36)	0.8573	1.15 (0.98 to 1.36)	0.0822
**5–9s**						
Month counter (A)	1.02 (1.02 to 1.02)	<0.0001*	1.02 (1.02 to 1.02)	<0.0001*	1.02 (1.02 to 1.02)	<0.0001*
COVID-19 flag (B)	3.97 (3.18 to 4.95)	<0.0001*	3.40 (2.50 to 4.62)	<0.0001*	4.76 (3.35 to 6.78)	<0.0001*
A×B	0.98 (0.94 to 1.02)	0.2519	0.98 (0.93 to 1.03)	0.4811	0.97 (0.91 to 1.03)	0.3034
**10–24s**						
Month counter (A)	1.02 (1.02 to 1.02)	<0.0001*	1.02 (1.02 to 1.03)	<0.0001*	1.02 (1.02 to 1.02)	<0.0001*
COVID-19 flag (B)	2.83 (2.43 to 3.30)	<0.0001*	2.89 (2.35 to 3.54)	<0.0001*	3.23 (2.47 to 4.22)	<0.0001*
A×B	1.03 (1.01 to 1.06)	0.0192*	1.03 (0.99 to 1.07)	0.1037	1.03 (0.98 to 1.08)	0.1833

*, p<0.05.

The overall appendicectomy rate of the last 3 pandemic months remained unchanged from the pre-pandemic rate for the under-5s (all: p=0.4711; simple: p=0.7544; complex: p=0.2873). There was a similar pattern for complex admissions (5–9s: p=0.1505; 10–24s: p=0.2238). Overall appendicectomy rates for simple appendicitis admissions significantly increased in the later pandemic months for the 5–9s (91.3% vs 94.5%, p=0.0159), and decreased for the 10–24s (95.3% vs 91.2%, p<0.0001).

### Laparoscopic appendicectomy

**Figure 2:**
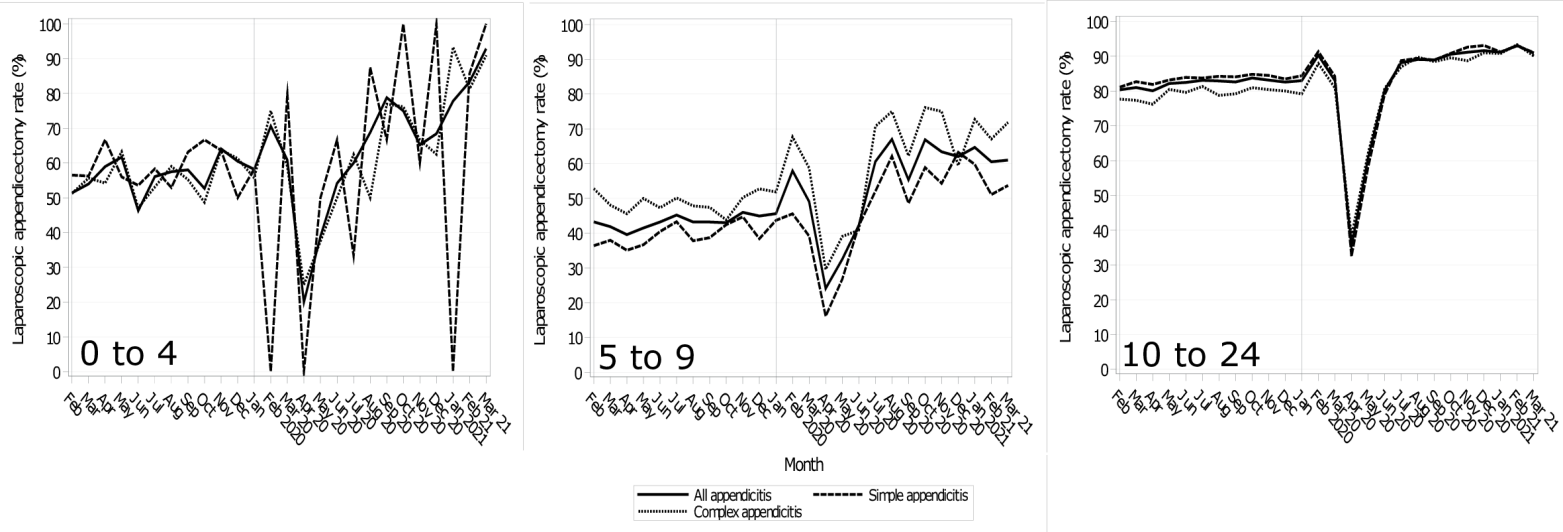
Monthly laparoscopic appendicectomy rate trends during the pandemic and the mean monthly rates for the previous 5 years, stratified by appendicitis type and age group.

There was a clear increasing pre-pandemic trend for laparoscopic appendicectomies, as opposed to an open appendicectomy, with approximately 85% performed laparoscopically in CYP before the pandemic (online supplemental figure 2). Laparoscopic appendicectomy rates fell sharply in April (under-5s all: −39.0% (−51.2 to −26.7); simple: −66.7% (−82.7 to −50.7); complex: −29.2% (−39.8 to −18.6); 5–9s all: −15.5% (−23.2 to −7.8); simple:−18.9% (−27.5 to −10.4); complex: −16.0% (−23.9 to −8.2)); 10–24s all: −44.8% (−57.9 to −31.6); simple:−49.5% (−63.3 to −35.7); complex: −37.9% (−49.9 to −25.8)) and May 2020, followed by a rapid recovery, across all subtypes and ages from July 2020 onwards (figure 2 and online supplemental tables 7–9). The largest absolute reductions were among the 10–24s for April 2020 (all: −643 procedures (−693 to −593); simple: −395 (−434 to −356); complex: 143 (−166 to −119)) (online supplemental tables 10–12).

After the sharp decline in laparoscopic appendicectomy rates early in the pandemic, rates rebounded significantly and peaked in March 2021 (all: +38.9% (26.7 to 51.1)) for the under-5s, October (all: +23.9% (14.3 to 33.4)) for the 5–9s and February 2021 (all: +12.7% (5.7 to 19.7)) for the 10–24s (online supplemental tables 7–9). The equivalent absolute difference was non-significant for the under-5s (+0.8 (−1.0 to 2.6)) and significant, but of relatively minor clinical effect, for the older age groups (5–9s: +28.0 (17.6 to 38.4); 10–24s: +10.0 (3.8 to 16.2)) (online supplemental tables 10–12).

The odds of laparoscopic appendicectomy significantly increased after the first wave for all admissions (under-5s: OR 2.53 (1.23 to 5.23), p=0.0118; 5–9s: 3.97 (3.18 to 4.95), p<0.0001; 10–24s: 2.83 (2.43 to 3.30), p<0.0001) (table 2). For the under-5s and 10–24s, the gradient of the laparoscopic appendicectomy rate did not significantly change, but for the 10–24s, the gradient was significantly but modestly steeper after the first wave for all appendicitis (OR 1.03 (1.01 to 1.06), p=0.0192).

Laparoscopic appendicectomies significantly increased in the last quarter, compared with the pre-pandemic period, across all age groups and subtypes, except for simple appendicitis among the under-5s (57.3% vs 69.2%, p=0.5691). Overall, laparoscopic appendicectomies for all (56.5% vs 83.9%, p<0.0001) and complex (55.7% vs 88.1%, p<0.0001) for the under-5s significantly increased in the last 3 pandemic months, compared with the pre-pandemic period. The 5–9s (all: 43.4% vs 61.9%, p<0.0001; simple: 39.7% vs 54.8%, p<0.0001 and complex: 49.0% vs 70.3%, p<0.0001) and 10–24s had similar trends (all: 82.3% vs 91.7%, p<0.0001; simple: 83.5% vs 91.7%, p<0.0001 and complex; 79.3% vs 91.4%, p<0.0001).

### Funnel plots

**Figure 3:**
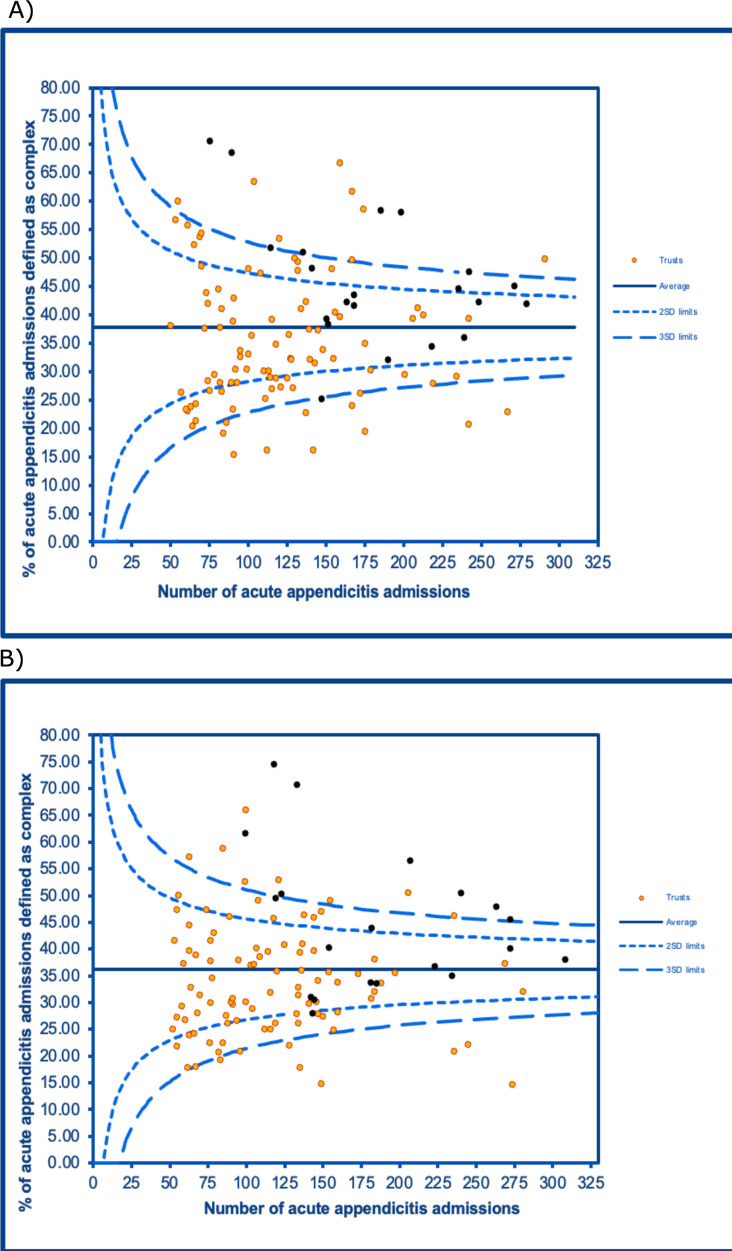
Funnel plot of (A) pre-pandemic and (B) pandemic crude complex appendicitis rates by trust in England, where hospital trusts with specialist paediatric surgical centres are marked black.

The funnel plots reflect 115 hospitals. 21 of the 22 hospitals with specialist paediatric surgical services (specialist) were included. The funnel plots showed significant variation in both pre-pandemic and pandemic rates (figure 3). Further details on the funnel plot are provided elsewhere (online supplemental text C).

### Sensitivity analysis

Sensitivity and main analysis definitions generated similar counts across age groups (online supplemental figures D–F).

## Discussion

There were fundamental changes in the care of CYP with appendicitis in the first 14 months of the COVID-19 pandemic. The proportion of CYP diagnosed with complex appendicitis increased, driven more by a reduction in simple rather than an increase in complex admissions, aligning with international studies spanning shorter periods.^28^ It is unclear why hospital admissions by CYP with simple appendicitis decreased. Possible reasons include earlier commencement of antibiotics before a confirmed diagnosis, changes to seasonal and helminth causes of appendicitis or spontaneous resolution of symptoms.^29^ During the pandemic, there was a small but significant increase in the proportion of complex appendicitis treated within hospitals with specialist paediatric surgical centres. Before the pandemic, there were variations in the quality of care for appendicitis, which were identified by the Getting It Right First Time report, including variations in outcomes depending on where complex appendicitis is managed.^9^ Previous efforts to standardise care for CYP with acute appendicitis have included the introduction of paediatric networks at regional and national levels to improve the coordination of surgical services, with some early signs of improvement.^9^

Like many other European countries,^30^ despite guidance advising against surgical procedures involving AGP, treatment of complex appendicitis remained relatively steady. More than 95% of complex appendicitis admissions for the 5–24s were treated with an appendicectomy. For the under-5s, who are more likely to present with complex appendicitis and not treated with an appendicectomy because of an appendix mass, the absolute difference in appendicectomies did not significantly change between periods for most pandemic months. This persistence in operative management throughout the pandemic demonstrates appendicectomies’ pivotal role in treating complex appendicitis.

There was a pre-pandemic reduction in the proportion of appendicitis admissions treated by appendicectomy, which adds to the growing international literature in adults^31 32^ and CYP^33^ on the trend towards non-operative management of simple appendicitis. In keeping with some of the literature,^16 34^ a significant decrease in appendicectomy for simple appendicitis was seen across all ages in the first pandemic wave, with up to 35% non-operatively managed. It is unknown if an even higher proportion of CYP with presumed simple appendicitis started and failed a trial of non-operative management, resulting in a delay to appendicectomy, or whether the majority of those managed with antibiotics were successfully treated. Interestingly, this trend did not continue after the first pandemic wave, and the odds of undergoing appendicectomy for simple appendicitis were equivalent to or higher than that before the pandemic. This highlights an important area for future research into operative and non-operative pathways for appendicitis management with careful investigation of the success rate and the risk of recurrent appendicitis and operative complications.

The pre-pandemic laparoscopic appendicectomy rate steadily increased such that over 85% of appendicectomies in CYP were performed laparoscopically. During the first wave, the guidance cautioned the use of laparoscopy due to concerns it may contribute to SARS-CoV-2 spread. This reflects our findings where, during the first pandemic wave, <40% of appendectomies were performed laparoscopically, matching a smaller study on adults.^35^ Reassuringly, this was rapidly reversed, and the pre-pandemic trend of increasing laparoscopic appendectomy rates for CYP has continued.

The provision of care for CYP with complex appendicitis changed during the first pandemic year. While paediatric surgical centres are normally more likely to care for a higher proportion of children with complex appendicitis, these findings suggest that even more CYP were transferred to specialist centres during the pandemic. This could reflect the diversion of paediatric healthcare staff and areas to adult care during the pandemic, particularly within non-specialist centres, or changes to operating theatre availability for CYP with complex appendicitis could suggest that the CYP with complex appendicitis during the pandemic were more unwell. A more detailed investigation of why there is hospital-level variation is beyond the scope of this paper, but these findings highlight the need for more in-depth studies, which can be supported through initiatives like national audit programmes. Future studies could investigate the effects of the pandemic on length of stay, negative appendicectomy rate and laparoscopic appendicectomy rate by hospitals with or without specialist paediatric services. Additionally, future research could investigate if paediatric surgical centres continued to care for a higher proportion of children with complex appendicitis beyond the first 14 months of the pandemic.

### Strengths and limitations

This study is the first to report a national overview of the month-by-month and overall trends and referral patterns spanning 14 months. First, this builds upon previous work focusing on the early pandemic weeks, adult populations or smaller groups of hospitals, whose findings cannot be generalised nationally. Incorporating 5 pre-pandemic years is a particular strength as it challenged assumptions of no pre-pandemic trends, highlighting the importance of accurately contextualising findings. Second, the large sample size provided robust numbers, facilitating stratifications by age and subtypes, and reporting of granular changes in relative and absolute terms. Third, applying ITS analysis, an advanced statistical methodology generates robust and more accurate results.^36^ Fourth, primary diagnosis and procedure codes in HES are >95%^37^ accurate, further supporting the validity of our findings. Fifth, the cohort study design is methodologically robust over case reports and cross-sectional studies. Sixth, the month-by-month findings empower national decision-makers with details on how hospitals coped with changes during the pandemic, which can inform responses to possible future outbreaks or pandemics.

Limitations were first, being unable to draw causal inferences due to unmeasured confounding. Second, HES does not offer linked prescribing, laboratory or physiological data, such as diagnostic imaging or antibiotic prescribing data. Third, it was not possible to know which admissions were confirmed by diagnostic imaging or how diagnostic imaging evolved during the pandemic. CT scans are rarely used to diagnose appendicitis in children, but what impact possible changes to diagnostic imaging over the pandemic on diagnosis remains unclear. Fourth, one single-centre study reported underestimation of complex appendicitis coding^36^; however, caution is needed before generalising results from a single-site study from 2012, nationally. Additionally, this study compared complex appendicitis between pandemic periods and found no indication that coding had changed suggesting they remained relatively consistent.

### Policy and clinical practice implications

The pandemic may have strengthened previous efforts for reducing variation in the management of complex appendicitis, given that three fewer non-specialist centres had positive outliers for complex appendicitis during the pandemic than before it. We found a reduction in the proportion of simple appendicitis admissions treated surgically during the pandemic, contributing to the wider debate on the role of surgery in the treatment of simple appendicitis. These findings can build upon the existing literature to inform future recommendations and thresholds for stopping laparoscopic surgeries in possible future pandemics for surgically managed acute appendicitis.

## Conclusions

Appendicectomy and laparoscopic appendicectomy rates recovered sharply after a steep drop in April 2020. These results provide national context to smaller studies reporting the immediate short-term impacts of COVID-19 appendicitis management.

## Data Availability

Data may be obtained from a third party and are not publicly available. All data relevant to the study are included in the article or uploaded as supplemental information. Data may be obtained from a third party and are not publicly available. All data relevant to the study are included in the article or uploaded as supplemental information. The pseudonymised patient data that were used for this study can be accessed by contacting NHS Digital (see https://digital.nhs.uk/services/data-access-request-service-dars). Access to these data is subject to a data sharing agreement (DSA) containing detailed terms and conditions of use following protocol approval from NHS Digital.
