# Supplementary material for: Impact of the COVID-19 pandemic on the clinical management trends for acute appendicitis among the under-25s: a retrospective study
Source: Arch Dis Child. 2024 Feb 7;109(4):339–46. doi: 10.1136/archdischild-2023-326313 (PMC10958286; doi:10.1136/archdischild-2023-326313)

Table of Contents

Appendicectomy rates *2*

Figure S1: Pre-pandemic three-monthly appendicectomy rate trend 2

Table S1: The percentage difference between the monthly appendicectomy rate during the pandemic and the five-year pre-pandemic monthly mean by the 5-9s 2

Table *S2*: The percentage difference between the monthly appendicectomy rate during the pandemic and the five-year pre-pandemic monthly mean by the 10-24s 3

Table S*3*: The absolute difference between the monthly appendicectomy counts during the pandemic and the five-year pre-pandemic monthly mean counts by the 5-9s 3

Table S4: The absolute difference between the monthly appendicectomy counts during the pandemic and the five-year pre-pandemic monthly mean counts by the 10-24s 4

Table S5: The percentage difference between the monthly appendicectomy rate during the pandemic and the five-year pre-pandemic monthly mean by the 0-4s 4

Table *S6*: The absolute difference between the monthly appendicectomy counts during the pandemic and the five-year pre-pandemic monthly mean counts by the 0-4s 5

Laparoscopic appendicectomy rates *6*

Figure S2: Pre-pandemic quarterly trends for laparoscopic appendicectomy rate 6

Table S7: The percentage difference between the monthly laparoscopic appendicectomy rate, by the 0-4s, during the pandemic and the monthly mean laparoscopic appendicectomy rate for the previous five years 6

Table S8: The percentage difference between the monthly laparoscopic appendicectomy rate, by the 5-9s, during the pandemic and the monthly mean laparoscopic appendicectomy rate for the previous five years 6

Table S9: The percentage difference between the monthly laparoscopic appendicectomy rate, by the 10-24s, during the pandemic and the monthly mean laparoscopic appendicectomy rate for the previous five years 7

Table S10: Absolute differences in monthly laparoscopic appendicectomy counts, by the 0-4s, between the pandemic and the monthly mean counts for the previous five years 8

Table S11: Absolute differences in monthly laparoscopic appendicectomy counts, by the 5-9s, between the pandemic and the monthly mean counts for the previous five years 8

Table S12: Absolute differences in monthly laparoscopic appendicectomy counts, by the 10-24s, between the pandemic and the monthly mean counts for the previous five years 9

Coding definitions *9*

Table S.A: Acute appendicitis ICD-10 definitions for the main and sensitivity analyses 9

Table S.B: Definition of appendicectomy and laparoscopic appendicectomy OPCS code definitions based on published literature 10

Text S.C: Further detail on funnel plots 10

Sensitivity *11*

Figure S.D: Main and sensitivity monthly appendicitis admissions, by 0-4s, during the pandemic and the mean monthly admission trends for the previous five years 11

Figure S.E: Main and sensitivity monthly appendicitis admissions, by 5-9s, during the pandemic and the mean monthly admission trends for the previous five years 11

Figure S.F: Main and sensitivity monthly appendicitis admissions, by 10-24 year old's, during the pandemic and the mean monthly admission trends for the previous five years 12

Appendicectomy rates

Figure S1: Pre-pandemic three-monthly appendicectomy rate trend

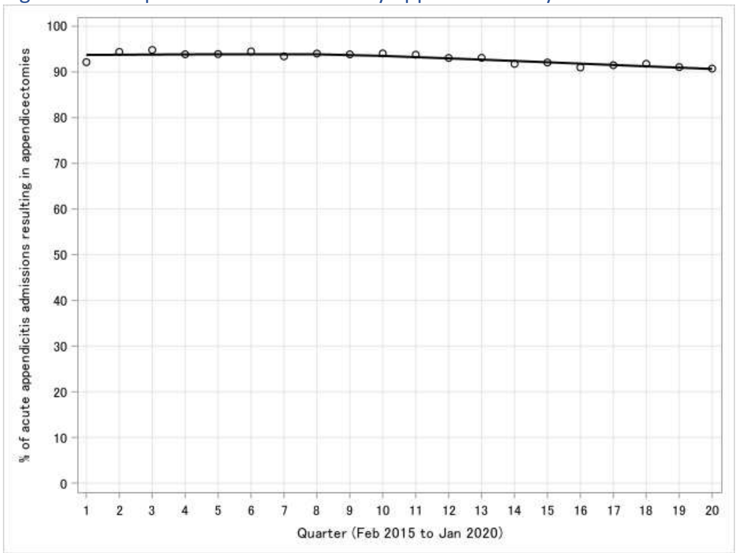

Table S1: The percentage difference between the monthly appendicectomy rate during the pandemic and the five-year pre-pandemic monthly mean by the 5-9s

	All			Simple			Complex		
	O	E	%Diff (95% CI)	O	E	%Diff (95% CI)	O	E	%Diff (95% CI)
Feb, 2020	90.1	93.7	-3.6 (-7.3 to 0.1)	89.8	95.8	-6.0 (-10.8 to -1.2)	97.0	96.3	0.7 (-0.9 to 2.4)
Mar	93.2	90.7	2.5 (-0.6 to 5.6)	94.9	94.8	0.1 (-0.5 to 0.7)	95.2	95.8	-0.6 (-2.1 to 0.9)
Apr	74.6	93.0	-18.4 (-26.8 to -10.0)	78.5	93.9	-15.4 (-23.1 to -7.7)	92.2	95.4	-3.2 (-6.7 to 0.3)
May	79.3	92.5	-13.2 (-20.3 to -6.1)	80.4	92.9	-12.5 (-19.4 to -5.6)	94.1	95.7	-1.6 (-4.1 to 0.9)
Jun	85.1	92.9	-7.8 (-13.3 to -2.4)	87.7	95.9	-8.2 (-13.9 to -2.6)	97.3	94.3	3 (-0.4 to 6.3)
Jul	85.2	93.2	-8.0 (-13.5 to -2.4)	89.1	95.2	-6.1 (-10.9 to -1.3)	93.4	93.5	-0.1 (-0.9 to 0.6)
Aug	86.4	93.2	-6.8 (-11.9 to -1.7)	90.4	94.9	-4.6 (-8.7 to -0.4)	91.7	95.1	-3.4 (-7.0 to 0.2)
Sep	88.8	93.0	-4.2 (-8.2 to -0.2)	91.5	94.6	-3.1 (-6.6 to 0.3)	94.7	97.1	-2.4 (-5.4 to 0.7)
Oct	90.7	95.0	-4.3 (-8.3 to -0.2)	94.7	96.5	-1.7 (-4.3 to 0.8)	98.5	96.5	2 (-0.8 to 4.8)
Nov	89.5	92.3	-2.8 (-6.1 to 0.5)	92.0	92.1	-0.1 (-0.5 to 0.4)	95.5	95.7	-0.1 (-0.9 to 0.6)
Dec	92.6	90.6	2.0 (-0.8 to 4.7)	96.1	93.9	2.2 (-0.7 to 5.1)	97.4	94.2	3.1 (-0.3 to 6.6)
Jan, 2021	89.8	91.7	-1.9 (-4.6 to 0.8)	91.1	93.2	-2.1 (-5.0 to 0.7)	97.1	96.0	1.1 (-1.0 to 3.2)

Feb	89.6	93.7	-4.1 (-8.1 to -0.1)	88.5	95.8	-7.3 (-12.6 to -2.0)	96.6	96.3	0.3 (-0.8 to 1.4)
Mar	93.5	90.7	2.8 (-0.5 to 6.0)	93.9	94.8	-0.9 (-2.7 to 1.0)	98.7	95.8	2.9 (-0.4 to 6.3)

Table *S2*: The percentage difference between the monthly appendicectomy rate during the pandemic and the five-year pre-pandemic monthly mean by the 10-24s

	All			Simple			Complex		
	O	E	%Diff (95% CI)	O	E	%Diff (95% CI)	O	E	%Diff (95% CI)
Feb, 2020	89.8	93.4	-3.6 (-7.3 to 0.1)	91.2	95.2	-4.0 (-7.9 to -0.1)	93.0	94.9	-1.9 (-4.6 to 0.8)
Mar	86.1	92.4	-6.3 (-11.2 to -1.4)	88.4	95.9	-7.5 (-12.9 to -2.2)	94.0	95.0	-1.0 (-3.0 to 1.0)
Apr	65.1	93.6	-28.4 (-38.9 to -18.0)	64.6	95.7	-31.1 (-42.1 to -20.2)	90.5	94.4	-3.9 (-7.7 to 0)
May	73.9	93.6	-19.7 (-28.5 to -11.0)	75.8	95.5	-19.6 (-28.3 to -10.9)	92.4	94.9	-2.6 (-5.7 to 0.6)
Jun	82.7	93.6	-10.9 (-17.4 to -4.5)	85.4	96.1	-10.7 (-17.1 to -4.3)	93.3	94.7	-1.4 (-3.6 to 0.9)
Jul	84.7	93.6	-8.9 (-14.7 to -3.1)	87.4	95.3	-7.9 (-13.4 to -2.4)	92.5	94.9	-2.4 (-5.5 to 0.6)
Aug	87.0	92.7	-5.6 (-10.3 to -1.0)	89.8	94.9	-5.1 (-9.5 to -0.7)	93.7	94.8	-1.1 (-3.2 to 1.0)
Sep	88.0	93.5	-5.5 (-10.1 to -0.9)	89.9	95.7	-5.8 (-10.5 to -1.1)	96.7	96.1	0.6 (-0.9 to 2.1)
Oct	90.7	93.1	-2.4 (-5.4 to 0.7)	92.4	95.3	-2.9 (-6.2 to 0.4)	97.2	96.0	1.2 (-0.9 to 3.4)
Nov	88.1	93.2	-5.1 (-9.6 to -0.7)	90.2	94.7	-4.5 (-8.6 to -0.3)	94.2	95.5	-1.3 (-3.5 to 0.9)
Dec	88.5	92.7	-4.2 (-8.2 to -0.2)	90.7	93.8	-3.1 (-6.5 to 0.4)	95.1	95.4	-0.3 (-1.2 to 0.7)
Jan, 2021	87.5	93.1	-5.6 (-10.2 to -0.9)	89.3	95.5	-6.1 (-11.0 to -1.3)	94.5	95.1	-0.6 (-2.2 to 0.9)
Feb	89.1	93.4	-4.3 (-8.4 to -0.2)	91.5	95.2	-3.7 (-7.4 to 0.1)	93.8	94.9	-1.1 (-3.3 to 0.9)
Mar	90.2	92.4	-2.2 (-5.1 to 0.7)	92.6	95.9	-3.3 (-6.8 to 0.3)	95.3	95.0	0.3 (-0.7 to 1.2)

Table S*3*: The absolute difference between the monthly appendicectomy counts during the pandemic and the five-year pre-pandemic monthly mean counts by the 5-9s

	All			Simple			Complex		
	O	E	Diff (95% CI)	O	E	Diff (95% CI)	O	E	Diff (95% CI)
Feb, 2020	164.0	191.0	-27.0 (-37.2 to -16.8)	79.0	91.2	-12.2 (-19 to -5.4)	65.0	77.4	-12.4 (-19.3 to -5.5)
Mar	165.0	203.0	-38.0 (-50.1 to -25.9)	74.0	98.0	-24 (-33.6 to -14.4)	80.0	78.4	1.6 (-0.9 to 4.0)
Apr	141.0	195.0	-54.0 (-68.4 to -39.6)	62.0	91.8	-29.8 (-40.5 to -19.1)	71.0	79.8	-8.8 (-14.7 to -3.0)
May	157.0	196.2	-39.2 (-51.5 to -26.9)	86.0	93.8	-7.8 (-13.3 to -2.3)	64.0	78.3	-14.3 (-21.8 to -6.9)
Jun	194.0	207.8	-13.8 (-21.1 to -6.5)	114.0	103.8	10.2 (3.9 to 16.5)	71.0	82.7	-11.7 (-18.4 to -5.0)
Jul	208.0	200.4	7.6 (2.2 to 13.0)	98.0	103.0	-5.0 (-9.4 to -0.6)	99.0	82.0	17.0 (8.9 to 25.1)
Aug	203.0	182.8	20.2 (11.4 to 29.0)	103.0	89.4	13.6 (6.4 to 20.8)	88.0	80.5	7.5 (2.1 to 12.9)
Sep	206.0	198.6	7.4 (2.1 to 12.7)	107.0	90.4	16.6 (8.6 to 24.6)	90.0	89.2	0.8 (-1.0 to 2.6)

Oct	166.0	193.0	-27.0 (-37.2 to -16.8)	90.0	92.8	-2.8 (-6.1 to 0.5)	67.0	78.5	-11.5 (-18.1 to -4.9)
Nov	153.0	196.6	-43.6 (-56.5 to -30.7)	81.0	98.0	-17.0 (-25.1 to -8.9)	64.0	73.7	-9.7 (-15.8 to -3.6)
Dec	187.0	181.6	5.4 (0.8 to 10.0)	98.0	82.8	15.2 (7.6 to 22.8)	74.0	79.0	-5.0 (-9.4 to -0.6)
Jan, 2021	167.0	203.0	-36.0 (-47.8 to -24.2)	92.0	96.6	-4.6 (-8.8 to -0.4)	66.0	83.0	-17.0 (-25.1 to -8.9)
Feb	190.0	191.0	-1.0 (-3.0 to 1.0)	92.0	91.2	0.8 (-1.0 to 2.6)	85.0	77.4	7.6 (2.2 to 13.0)
Mar	200.0	203.0	-3.0 (-6.4 to 0.4)	108.0	98.0	10.0 (3.8 to 16.2)	78.0	78.4	-0.4 (-1.7 to 0.9)

Table S4: The absolute difference between the monthly appendicectomy counts during the pandemic and the five-year pre-pandemic monthly mean counts by the 10-24s

	All			Simple			Complex		
	O	E	Diff (95% CI)	O	E	Diff (95% CI)	O	E	Diff (95% CI)
Feb, 2020	864.0	1011.8	-147.8 (-171.6 to -124)	521.0	587.6	-66.6 (-82.6 to -50.6)	240.0	268.3	-28.3 (-38.7 to -17.9)
Mar	757.0	1098.4	-341.4 (-377.6 to -305.2)	418.0	637.8	-219.8 (-248.9 to -190.7)	251.0	289.7	-38.7 (-50.9 to -26.5)
Apr	521.0	1032.6	-511.6 (-555.9 to -467.3)	275.0	591.2	-316.2 (-351.1 to -281.3)	219.0	284.5	-65.5 (-81.4 to -49.6)
May	715.0	1139.4	-424.4 (-464.8 to -384)	405.0	675.2	-270.2 (-302.4 to -238)	254.0	299.5	-45.5 (-58.7 to -32.3)
Jun	932.0	1143.0	-211 (-239.5 to -182.5)	560.0	649.2	-89.2 (-107.7 to -70.7)	307.0	334.8	-27.8 (-38.2 to -17.5)
Jul	936.0	1068.8	-132.8 (-155.4 to -110.2)	569.0	611.4	-42.4 (-55.2 to -29.6)	283.0	314.8	-31.8 (-42.9 to -20.8)
Aug	952.0	1001.4	-49.4 (-63.2 to -35.6)	556.0	574.6	-18.6 (-27.1 to -10.1)	312.0	292.8	19.2 (10.6 to 27.7)
Sep	1031.0	1103.4	-72.4 (-89.1 to -55.7)	604.0	640.4	-36.4 (-48.2 to -24.6)	347.0	312.7	34.3 (22.8 to 45.8)
Oct	1094.0	1063.4	30.6 (19.8 to 41.4)	655.0	616.0	39.0 (26.8 to 51.2)	345.0	312.3	32.7 (21.5 to 43.9)
Nov	995.0	1105.0	-110 (-130.6 to -89.4)	596.0	640.8	-44.8 (-57.9 to -31.7)	310.0	301.0	9.0 (3.1 to 14.9)
Dec	930.0	985.8	-55.8 (-70.4 to -41.2)	536.0	552.8	-16.8 (-24.8 to -8.8)	311.0	297.8	13.2 (6.1 to 20.3)
Jan, 2021	938.0	1163.6	-225.6 (-255 to -196.2)	536.0	666.6	-130.6 (-153.0 to -108.2)	326.0	326.3	-0.3 (-1.5 to 0.8)
Feb	884.0	1011.8	-127.8 (-150 to -105.6)	529.0	587.6	-58.6 (-73.6 to -43.6)	285.0	268.3	16.7 (8.7 to 24.7)
Mar	987.0	1098.4	-111.4 (-132.1 to -90.7)	592.0	637.8	-45.8 (-59.1 to -32.5)	303.0	289.7	13.3 (6.1 to 20.4)

Table S5: The percentage difference between the monthly appendicectomy rate during the pandemic and the five-year pre-pandemic monthly mean by the 0-4s

	All			Simple			Complex		
	O	E	% Diff (95% CI)	O	E	% Diff (95% CI)	O	E	% Diff (95% CI)
Feb, 2020	73.9	89.0	-15.1 (-22.7 to -7.5)	50.0	88.5	-38.5 (-50.6 to -26.3)	88.9	94.9	-6.0 (-10.8 to -1.2)
Mar	82.1	83.7	-1.6 (-4.0 to 0.9)	71.4	71.4	0.0 (0.0 to 0.0)	94.4	92.1	2.4 (-0.6 to 5.4)

Apr	83.3	88.0	-4.6 (-8.9 to -0.4)	66.7	83.3	-16.7 (-24.7 to -8.7)	92.3	94.1	-1.8 (-4.3 to 0.8)
May	78.3	78.1	0.2 (-0.6 to 0.9)	33.3	67.6	-34.2 (-45.7 to -22.8)	100.0	87.2	12.8 (5.8 to 19.9)
Jun	77.4	84.0	-6.6 (-11.6 to -1.6)	60.0	77.8	-17.8 (-26 to -9.5)	95.2	91.5	3.8 (0.0 to 7.6)
Jul	71.4	83.1	-11.7 (-18.4 to -5.0)	60.0	81.8	-21.8 (-31 to -12.7)	72.7	88.2	-15.5 (-23.2 to -7.7)
Aug	69.6	82.2	-12.6 (-19.6 to -5.7)	100.0	85.0	15.0 (7.4 to 22.6)	72.7	87.5	-14.8 (-22.3 to -7.2)
Sep	86.8	86.2	0.7 (-0.9 to 2.3)	100.0	90.5	9.5 (3.5 to 15.6)	89.7	90.3	-0.6 (-2.2 to 0.9)
Oct	77.8	87.5	-9.7 (-15.8 to -3.6)	83.3	84.6	-1.3 (-3.5 to 0.9)	80.8	92.4	-11.6 (-18.3 to -4.9)
Nov	79.3	78.6	0.7 (-0.9 to 2.3)	71.4	84.6	-13.2 (-20.3 to -6.1)	100.0	91.6	8.4 (2.7 to 14.1)
Dec	86.4	82.1	4.3 (0.2 to 8.4)	60.0	73.3	-13.3 (-20.5 to -6.2)	94.1	92.4	1.7 (-0.8 to 4.3)
Jan, 2021	75.0	85.3	-10.3 (-16.6 to -4.0)	75.0	75.0	0.0 (0.0 to 0.0)	83.3	91.3	-8.0 (-13.4 to -2.4)
Feb	88.9	89.0	-0.1 (-0.7 to 0.5)	87.5	88.5	-1.0 (-2.9 to 1.0)	94.1	94.9	-0.8 (-2.5 to 1.0)
Mar	77.8	83.7	-5.9 (-10.7 to -1.2)	60.0	71.4	-11.4 (-18.1 to -4.8)	84.6	92.1	-7.4 (-12.8 to -2.1)

Table *S6*: The absolute difference between the monthly appendicectomy counts during the pandemic and the five-year pre-pandemic monthly mean counts by the 0-4s

	All			Simple			Complex		
	O	E	Diff (95% CI)	O	E	Diff (95% CI)	O	E	Diff (95% CI)
Feb, 2020	17.0	21.0	-4.0 (-7.9 to -0.1)	Supp*	4.6	Supp* (-7.3 to 0.1)	16.0	16.0	0.0 (0.0 to 0.0)
Mar	23.0	22.6	0.4 (-0.8 to 1.6)	5.0	4.0	1.0 (-1.0 to 3.0)	17.0	16.6	0.4 (-0.9 to 1.7)
Apr	15.0	23.4	-8.4 (-14.1 to -2.7)	Supp*	6.0	Supp* (-7.9 to -0.1)	12.0	15.8	-3.8 (-7.7 to 0.0)
May	18.0	21.4	-3.4 (-7.0 to 0.2)	Supp*	5.0	Supp* (-6.4 to 0.4)	16.0	15.8	0.2 (-0.6 to 1.0)
Jun	24.0	24.2	-0.2 (-1.1 to 0.7)	Supp*	5.6	Supp* (-5.8 to 0.6)	20.0	17.8	2.2 (-0.7 to 5.1)
Jul	20.0	24.6	-4.6 (-8.8 to -0.4)	Supp*	7.2	Supp*(-8.2 to -0.2)	16.0	16.2	-0.2 (-1.0 to 0.6)
Aug	16.0	24.0	-8.0 (-13.5 to -2.5)	8.0	6.8	1.2 (-0.9 to 3.3)	8.0	15.2	-7.2 (-12.4 to -1.9)
Sep	33.0	22.4	10.6 (4.2 to 17.0)	Supp*	7.6	Supp*(-8.8 to -0.4)	26.0	15.5	10.5 (4.1 to 16.9)
Oct	28.0	22.4	5.6 (1.0 to 10.2)	5.0	6.6	-1.6 (-4.1 to 0.9)	21.0	16.2	4.8 (0.5 to 9.1)
Nov	23.0	20.6	2.4 (-0.6 to 5.4)	5.0	4.4	0.6 (-0.9 to 2.1)	18.0	16.3	1.7 (-0.9 to 4.2)
Dec	19.0	23.8	-4.8 (-9.1 to -0.5)	Supp*	4.4	Supp* (-3.7 to 0.9)	16.0	18.2	-2.2 (-5.1 to 0.7)
Jan, 2021	18.0	22.0	-4.0 (-7.9 to -0.1)	Supp*	4.8	Supp* (-4.4 to 0.8)	15.7	15.0	-0.7 (-2.3 to 0.9)
Feb	24.0	21.0	3.0 (-0.4 to 6.4)	7.0	4.6	2.4 (-0.6 to 5.4)	16.0	16.0	0.0 (0.0 to 0.0)
Mar	14.0	22.6	-8.6 (-14.3 to -2.9)	Supp*	4.0	Supp* (-3.0 to 1.0)	11.0	16.6	-5.6 (-10.2 to -0.9)

Laparoscopic appendicectomy rates

Figure S2: Pre-pandemic quarterly trends for laparoscopic appendicectomy rate

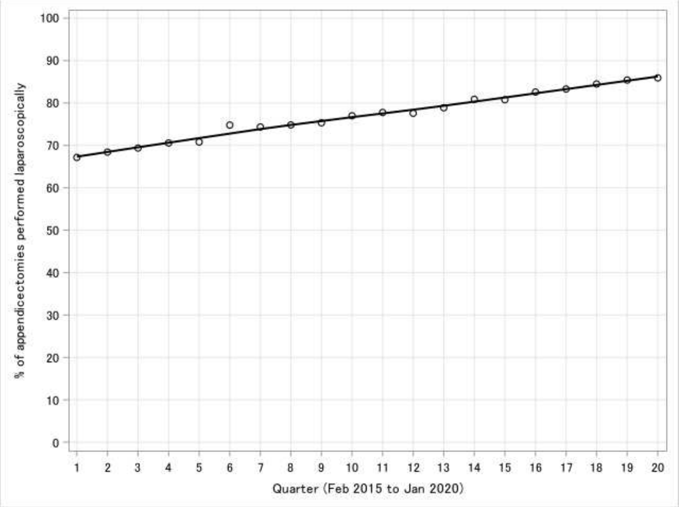

Table S7: The percentage difference between the monthly laparoscopic appendicectomy rate, by the 0-4s, during the pandemic and the monthly mean laparoscopic appendicectomy rate for the previous five years

	All			Simple			Complex		
	O	E	% Diff (95% CI)	O	E	% Diff (95% CI)	O	E	% Diff (95% CI)
Feb, 2020	70.6	51.4	19.2 (10.6 to 27.7)	0.0	56.5	-56.5 (-71.3 to -41.8)	75.0	51.3	23.8 (14.2 to 33.3)
Mar	60.9	54.0	6.9 (1.7 to 12.0)	80.0	56.3	23.8 (14.2 to 33.3)	58.8	55.7	3.1 (-0.3 to 6.6)
Apr	20.0	59.0	-39.0 (-51.2 to -26.7)	0.0	66.7	-66.7 (-82.7 to -50.7)	25.0	54.2	-29.2 (-39.8 to -18.6)
May	38.9	61.7	-22.8 (-32.2 to -13.4)	50.0	56.0	-6.0 (-10.8 to -1.2)	37.5	63.3	-25.8 (-35.7 to -15.8)
Jun	54.2	46.3	7.9 (2.4 to 13.4)	66.7	53.6	13.1 (6.0 to 20.2)	50.0	47.1	2.9 (-0.4 to 6.2)
Jul	60.0	56.1	3.9 (0.0 to 7.8)	33.3	58.3	-25.0 (-34.8 to -15.2)	62.5	53.1	9.4 (3.4 to 15.4)
Aug	68.8	57.5	11.3 (4.7 to 17.8)	87.5	52.9	34.6 (23.0 to 46.1)	50.0	59.0	-9.0 (-14.9 to -3.1)
Sep	78.8	58.0	20.8 (11.8 to 29.7)	66.7	63.2	3.5 (-0.2 to 7.2)	76.9	55.2	21.7 (12.6 to 30.8)
Oct	75.0	52.7	22.3 (13.1 to 31.6)	100.0	66.7	33.3 (22.0 to 44.6)	76.2	48.7	27.5 (17.2 to 37.8)
Nov	65.2	64.1	1.1 (-1.0 to 3.2)	60.0	63.6	-3.6 (-7.4 to 0.1)	66.7	63.8	2.9 (-0.4 to 6.3)
Dec	68.4	60.5	7.9 (2.4 to 13.4)	100.0	50.0	50.0 (36.1 to 63.9)	62.5	61.3	1.2 (-0.9 to 3.4)
Jan, 2021	77.8	58.2	19.6 (10.9 to 28.3)	0.0	58.3	-58.3 (-73.3 to -43.4)	93.3	55.7	37.6 (25.6 to 49.7)
Feb	83.3	51.4	31.9 (20.8 to 43)	85.7	56.5	29.2 (18.6 to 39.8)	81.3	51.3	30.0 (19.3 to 40.7)
Mar	92.9	54.0	38.9 (26.7 to 51.1)	100.0	56.3	43.8 (30.8 to 56.7)	90.9	55.7	35.2 (23.6 to 46.9)

Table S8: The percentage difference between the monthly laparoscopic appendicectomy rate, by the 5-9s, during the pandemic and the monthly mean laparoscopic appendicectomy rate for the previous five years

	All			Simple			Complex		
	O	E	% Diff (95% CI)	O	E	% Diff (95% CI)	O	E	% Diff (95% CI)

Feb, 2020	57.9	43.2	14.7 (7.2 to 22.2)	45.6	36.4	9.2 (3.2 to 15.1)	67.7	52.8	14.9 (7.3 to 22.4)
Mar	49.1	41.9	7.2 (2.0 to 12.5)	39.2	38.0	1.2 (-0.9 to 3.4)	58.8	48.1	10.7 (4.3 to 17.1)
Apr	24.1	39.6	-15.5 (-23.2 to -7.8)	16.1	35.1	-18.9 (-27.5 to -10.4)	29.6	45.6	-16.0 (-23.9 to -8.2)
May	32.5	41.5	-9.0 (-14.9 to -3.1)	26.7	36.7	-9.9 (-16.1 to -3.8)	39.1	50.0	-10.9 (-17.4 to -4.5)
Jun	42.3	43.2	-0.9 (-2.9 to 1)	42.1	40.5	1.6 (-0.9 to 4.2)	40.8	47.3	-6.4 (-11.4 to -1.5)
Jul	60.6	45.2	15.4 (7.7 to 23.1)	52.0	43.3	8.7 (2.9 to 14.5)	70.7	50.1	20.6 (11.7 to 29.5)
Aug	67.0	43.2	23.8 (14.2 to 33.3)	62.1	37.8	24.3 (14.7 to 34)	75.0	47.8	27.2 (16.9 to 37.4)
Sep	55.3	43.2	12.1 (5.3 to 19)	48.6	38.7	9.9 (3.7 to 16)	62.2	47.4	14.8 (7.3 to 22.3)
Oct	66.9	43.0	23.9 (14.3 to 33.4)	58.9	42.5	16.4 (8.5 to 24.4)	76.1	43.8	32.3 (21.2 to 43.4)
Nov	63.4	46.0	17.4 (9.2 to 25.6)	54.3	44.7	9.6 (3.5 to 15.7)	75.0	50.3	24.7 (15 to 34.5)
Dec	62.0	44.9	17.1 (9.0 to 25.2)	63.3	38.4	24.9 (15.1 to 34.6)	59.5	52.8	6.7 (1.6 to 11.8)
Jan, 2021	64.7	45.6	19.1 (10.5 to 27.6)	59.8	43.7	16.1 (8.2 to 24.0)	72.7	51.9	20.9 (11.9 to 29.8)
Feb	60.5	43.2	17.3 (9.1 to 25.4)	51.1	36.4	14.7 (7.2 to 22.2)	67.1	52.8	14.3 (6.9 to 21.7)
Mar	61.0	41.9	19.1 (10.6 to 27.7)	53.7	38.0	15.7 (8.0 to 23.5)	71.8	48.1	23.7 (14.2 to 33.3)

Table S9: The percentage difference between the monthly laparoscopic appendicectomy rate, by the 10-24s, during the pandemic and the monthly mean laparoscopic appendicectomy rate for the previous five years

	All			Simple			Complex		
	O	E	% Diff (95% CI)	O	E	% Diff (95% CI)	O	E	% Diff (95% CI)
Feb, 2020	90.3	80.4	9.9 (3.8 to 16.1)	91.4	81.1	10.3 (4.0 to 16.5)	87.9	77.7	10.2 (4.0 to 16.5)
Mar	83.0	81.0	2.0 (-0.8 to 4.7)	84.2	82.7	1.5 (-0.9 to 3.9)	80.9	77.3	3.5 (-0.1 to 7.2)
Apr	35.3	80.1	-44.8 (-57.9 to -31.6)	32.4	81.9	-49.5 (-63.3 to -35.7)	38.4	76.2	-37.9 (-49.9 to -25.8)
May	60.7	82.2	-21.5 (-30.5 to -12.4)	58.0	83.2	-25.2 (-35.0 to -15.3)	62.2	80.5	-18.3 (-26.7 to -9.9)
Jun	80.3	82.5	-2.2 (-5.1 to 0.7)	78.9	83.9	-5.0 (-9.4 to -0.6)	80.8	79.6	1.2 (-1.0 to 3.3)
Jul	88.1	83.1	5.1 (0.6 to 9.5)	88.8	83.7	5.0 (0.6 to 9.4)	86.9	81.3	5.6 (1.0 to 10.2)
Aug	89.1	82.9	6.2 (1.3 to 11.0)	89.4	84.2	5.2 (0.7 to 9.6)	89.7	78.8	11.0 (4.5 to 17.5)
Sep	88.9	82.6	6.3 (1.4 to 11.3)	88.9	84.1	4.8 (0.5 to 9.1)	88.5	79.2	9.3 (3.3 to 15.2)
Oct	90.6	83.8	6.8 (1.7 to 12.0)	90.8	84.8	6.0 (1.2 to 10.8)	89.6	81.0	8.6 (2.9 to 14.3)
Nov	91.2	83.1	8.0 (2.5 to 13.6)	92.6	84.5	8.1 (2.5 to 13.7)	88.7	80.4	8.3 (2.7 to 13.9)
Dec	91.6	82.6	9.0 (3.1 to 14.9)	93.1	83.5	9.6 (3.5 to 15.7)	91.0	80.0	11.0 (4.5 to 17.5)
Jan, 2021	91.2	83.0	8.2 (2.6 to 13.7)	91.2	84.4	6.8 (1.7 to 12.0)	90.8	79.2	11.6 (4.9 to 18.3)
Feb	93.1	80.4	12.7 (5.7 to 19.7)	93.0	81.1	11.9 (5.1 to 18.7)	93.3	77.7	15.7 (7.9 to 23.4)
Mar	90.9	81.0	9.9 (3.7 to 16.0)	91.0	82.7	8.4 (2.7 to 14.0)	90.1	77.3	12.8 (5.8 to 19.8)

Table S10: Absolute differences in monthly laparoscopic appendicectomy counts, by the 0-4s, between the pandemic and the monthly mean counts for the previous five years

	All			Simple			Complex		
	O	E	Diff (95% CI)	O	E	Diff (95% CI)	O	E	Diff (95% CI)
Feb, 2020	12.0	10.8	1.2 (-0.9 to 3.3)	Supp*	2.6	Supp*(-0.8 to 4.3)	12.0	8.2	3.8 (0.0 to 7.6)
Mar	14.0	12.2	1.8 (-0.8 to 4.4)	Supp*	2.3	Supp*(-0.8 to 4.3)	10.0	9.8	0.2 (-0.7 to 1.1)
Apr	Supp*	13.8	Supp*(-17.2 to -4.4)	Supp*	4.0	Supp*(0.1 to 7.9)	Supp*	9.0	Supp*(-10.8 to -1.2)
May	7.0	13.2	-6.2 (-11.1 to -1.3)	Supp*	2.8	Supp*(-4.4 to 0.8)	6.0	10.0	-4.0 (-7.9 to -0.1)
Jun	13.0	11.2	1.8 (-0.8 to 4.4)	Supp*	3.0	Supp*(-3.0 to 1.0)	10.0	8.2	1.8 (-0.8 to 4.4)
Jul	12.0	13.8	-1.8 (-4.4 to 0.8)	Supp*	4.2	Supp*(-6.7 to 0.3)	10.0	8.6	1.4 (-0.9 to 3.7)
Aug	11.0	13.8	-2.8 (-6.1 to 0.5)	7.0	3.6	3.4 (-0.2 to 7.0)	Supp*	9.8	Supp*(-10.5 to -1.1)
Sep	26.0	13.0	13.0 (5.9 to 20.1)	Supp*	4.8	Supp*(-6.1 to 0.5)	20.0	7.4	12.6 (5.6 to 19.6)
Oct	21.0	11.8	9.2 (3.3 to 15.1)	5.0	4.4	0.6 (-0.9 to 2.1)	16.0	7.4	8.6 (2.9 to 14.3)
Nov	15.0	13.2	1.8 (-0.8 to 4.4)	Supp*	2.8	Supp*(-0.7 to 1.1)	12.0	10.2	1.8 (-0.8 to 4.4)
Dec	13.0	14.4	-1.4 (-3.7 to 0.9)	Supp*	2.2	Supp*(-1 to 2.6)	10.0	11.4	-1.4 (-3.7 to 0.9)
Jan, 2021	14.0	12.8	1.2 (-0.9 to 3.3)	Supp*	2.8	Supp*(-0.5 to 6.1)	14.0	8.8	5.2 (0.7 to 9.7)
Feb	20.0	10.8	9.2 (3.3 to 15.1)	6.0	2.6	3.4 (-0.2 to 7)	13.0	8.2	4.8 (0.5 to 9.1)
Mar	13.0	12.2	0.8 (-1.0 to 2.6)	Supp*	2.3	0.8 (-0.9 to 2.4)	10.0	9.8	0.2 (-0.7 to 1.1)

Table S11: Absolute differences in monthly laparoscopic appendicectomy counts, by the 5-9s, between the pandemic and the monthly mean counts for the previous five years

	All			Simple			Complex		
	O	E	Diff (95% CI)	O	E	Diff (95% CI)	O	E	Diff (95% CI)
Feb, 2020	95.0	82.6	12.4 (5.5 to 19.3)	36.0	33.2	2.8 (-0.5 to 6.1)	44.0	41.4	2.6 (-0.6 to 5.8)
Mar	81.0	85.0	-4.0 (-7.9 to -0.1)	29.0	37.2	-8.2 (-13.8 to -2.6)	47.0	37.6	9.4 (3.4 to 15.4)
Apr	34.0	77.2	-43.2 (-56.1 to -30.3)	10.0	32.2	-22.2 (-31.4 to -13.0)	21.0	37.2	-16.2 (-24.1 to -8.3)
May	51.0	81.4	-30.4 (-41.2 to -19.6)	23.0	34.4	-11.4 (-18.0 to -4.8)	25.0	40.6	-15.6 (-23.3 to -7.9)
Jun	82.0	89.8	-7.8 (-13.3 to -2.3)	48.0	42.0	6.0 (1.2 to 10.8)	29.0	40.2	-11.2 (-17.8 to -4.6)
Jul	126.0	90.6	35.4 (23.7 to 47.1)	51.0	44.6	6.4 (1.4 to 11.4)	70.0	39.4	30.6 (19.8 to 41.4)
Aug	136.0	79.0	57.0 (42.2 to 71.8)	64.0	33.8	30.2 (19.4 to 41)	66.0	37.8	28.2 (17.8 to 38.6)
Sep	114.0	85.8	28.2 (17.8 to 38.6)	52.0	35.0	17.0 (8.9 to 25.1)	56.0	42.2	13.8 (6.5 to 21.1)

Oct	111.0	83.0	28.0 (17.6 to 38.4)	53.0	39.4	13.6 (6.4 to 20.8)	51.0	35.4	15.6 (7.9 to 23.3)
Nov	97.0	90.4	6.6 (1.6 to 11.6)	44.0	43.8	0.2 (-0.7 to 1.1)	48.0	38.0	10.0 (3.8 to 16.2)
Dec	116.0	81.6	34.4 (22.9 to 45.9)	62.0	31.8	30.2 (19.4 to 41.0)	44.0	42.2	1.8 (-0.8 to 4.4)
Jan, 2021	108.0	92.6	15.4 (7.7 to 23.1)	55.0	42.2	12.8 (5.8 to 19.8)	48.0	44.8	3.2 (-0.3 to 6.7)
Feb	115.0	82.6	32.4 (21.2 to 43.6)	47.0	33.2	13.8 (6.5 to 21.1)	57.0	41.4	15.6 (7.9 to 23.3)
Mar	122.0	85.0	37.0 (25.1 to 48.9)	58.0	37.2	20.8 (11.9 to 29.7)	56.0	37.6	18.4 (10 to 26.8)

Table S12: Absolute differences in monthly laparoscopic appendicectomy counts, by the 10-24s, between the pandemic and the monthly mean counts for the previous five years

	All			Simple			Complex		
	O	E	Diff (95% CI)	O	E	Diff (95% CI)	O	E	Diff (95% CI)
Feb, 2020	780.0	813.0	-33.0 (-44.3 to -21.7)	476.0	476.6	-0.6 (-2.1 to 0.9)	211.0	210.2	0.8 (-1.0 to 2.6)
Mar	628.0	889.8	-261.8 (-293.5 to -230.1)	352.0	527.4	-175.4 (-201.4 to -149.4)	203.0	228.0	-25.0 (-34.8 to -15.2)
Apr	184.0	826.8	-642.8 (-692.5 to -593.1)	89.0	484.2	-395.2 (-434.2 to -356.2)	84.0	226.8	-142.8 (-166.2 to -119.4)
May	434.0	936.2	-502.2 (-546.1 to -458.3)	235.0	561.6	-326.6 (-362 to -291.2)	158.0	248.4	-90.4 (-109 to -71.8)
Jun	748.0	942.6	-194.6 (-221.9 to -167.3)	442.0	544.8	-102.8 (-122.7 to -82.9)	248.0	271.0	-23 (-32.4 to -13.6)
Jul	825.0	888.0	-63.0 (-78.6 to -47.4)	505.0	512.0	-7.0 (-12.2 to -1.8)	246.0	261.2	-15.2 (-22.8 to -7.6)
Aug	848.0	830.2	17.8 (9.5 to 26.1)	497.0	484.0	13.0 (5.9 to 20.1)	280.0	227.6	52.4 (38.2 to 66.6)
Sep	917.0	911.6	5.4 (0.8 to 10.0)	537.0	538.4	-1.4 (-3.7 to 0.9)	307.0	242.2	64.8 (49.0 to 80.6)
Oct	991.0	890.6	100.4 (80.8 to 120)	595.0	522.4	72.6 (55.9 to 89.3)	309.0	247.6	61.4 (46.0 to 76.8)
Nov	907.0	918.8	-11.8 (-18.5 to -5.1)	552.0	541.4	10.6 (4.2 to 17.0)	275.0	240.6	34.4 (22.9 to 45.9)
Dec	852.0	814.0	38.0 (25.9 to 50.1)	499.0	461.4	37.6 (25.6 to 49.6)	283.0	236.2	46.8 (33.4 to 60.2)
Jan, 2021	855.0	965.8	-110.8 (-131.4 to -90.2)	489.0	562.6	-73.6 (-90.4 to -56.8)	296.0	258.4	37.6 (25.6 to 49.6)
Feb	823.0	813.0	10.0 (3.8 to 16.2)	492.0	476.6	15.4 (7.7 to 23.1)	266.0	210.2	55.8 (41.2 to 70.4)
Mar	897.0	889.8	7.2 (1.9 to 12.5)	539.0	527.4	11.6 (4.9 to 18.3)	273.0	228.0	45.0 (31.9 to 58.1)

Coding definitions

Table S.A: Acute appendicitis ICD-10 definitions for the main and sensitivity analyses

Analyses	ICD-10	Description
**Main**		
All	K35[1,2]	All acute appendicitis
	K37[1–3]	Unspecified appendicitis
Simple	K35.8	Acute appendicitis, other and unspecified

Complex	K35.2	Acute appendicitis with generalised peritonitis
	K35.3	Acute appendicitis with local peritonitis
**Sensitivity**		
All	K35[2]	All acute appendicitis
	K36[2,4]	Other appendicitis
	K37[2,4]	Unspecified appendicitis
	K38[4]	Other diseases of appendix

Table S.B: Definition of appendicectomy and laparoscopic appendicectomy OPCS code definitions based on published literature

OPCS	Description
**Appendicectomy**	
H011[2,3,5,6]	Emergency excision of appendix, Emergency excision of abnormal appendix and drainage hfq
H012[2,3,5,6]	Emergency excision of appendix, Emergency excision of abnormal appendix nec
H013[3–6]	Emergency excision of appendix, Emergency excision of normal appendix
H018[2–6]	Emergency excision of appendix, Other specified
H019[2,3,5,6]	Emergency excision of appendix, Unspecified
H028[2,3]	Other excision of appendix, Other specified
H029[2,3]	Other excision of appendix, Unspecified
**Laparoscopic appendicectomy**	
Y751[5]	Laparoscopically assisted approach to abdominal cavity
Y752[5]	Laparoscopic approach to abdominal cavity NEC

References:

1. Getting It Right First Time (GIRFT). Getting It Right First Time (GIRFT) Best Practice Library [Internet]. 2021. Available from: https://www.gettingitrightfirsttime.co.uk/bpl/
2. Zingone F, Sultan AA, Humes DJ, West J. Risk of Acute Appendicitis in and Around Pregnancy: A Population-based Cohort Study From England. Ann Surg [Internet]. 2015;261(2). Available from: https://journals.lww.com/annalsofsurgery/Fulltext/2015/02000/Risk_of_Acute_Appendicitis_in_and_Around.19.aspx
3. Al-Khyatt W, Mytton J, Tan BHL, Aquina CT, Evison F, Fleming FJ, et al. A Population-Based Cohort Study of Emergency Appendectomy Performed in England and New York State. World J Surg [Internet]. 2017;41(8):1975–84. Available from: https://doi.org/10.1007/s00268-017-3981-z
4. Faiz O, Clark J, Brown T, Bottle A, Antoniou A, Farrands P, et al. Traditional and laparoscopic appendectomy in adults: outcomes in English NHS hospitals between 1996 and 2006. Ann Surg. 2008 Nov;248(5):800–6.
5. Giuliani S, Cecil E V, Apelt N, Sharland M, Saxena S. Pediatric Emergency Appendectomy and 30-Day Postoperative Outcomes in District General Hospitals and Specialist Pediatric Surgical Centers in England, April 2001 to March 2012: Retrospective Cohort Study. Ann Surg [Internet]. 2016;263(1). Available from: https://journals.lww.com/annalsofsurgery/Fulltext/2016/01000/Pediatric_Emergency_Appendectomy_and_30_Day.27.aspx
6. Tanner S. Trends in children's surgery in England. Arch Dis Child [Internet]. 2007 Aug 1;92(8):664–7. Available from: http://adc.bmj.com/content/92/8/664.abstract

Text S.C: Further detail on funnel plots

20 hospitals comprising 1062 (6.5%) and 22 hospitals comprising 645 admissions (4.1%) were excluded in the pre-pandemic and pandemic period, respectively.

In Fig. 3A., 12 (10.4%) hospitals fell above and 19 (16.5%) fell below 2SD, while 12 (10.4%) hospitals fell above and 14 (12.2%) fell below 3SD. Among the hospitals with specialist paediatric services, 10 (47.6%) hospitals fell above and 1 (4.8%) fell below 2SD. For the same hospitals, 6 (28.6%) hospitals fell above and 1 (4.8%) fell

below 3SD. In Fig. 3B., 9 (7.8%) hospitals fell above and 18 (15.7%) fell below 2SD. 9 (7.8%) hospitals fell above and 16 (13.9%) fell below 3SD. Among the hospitals with specialist paediatric services, 11 (52.4%) and 1 (4.8%) hospital fell above and below the 2SD control limit respectively, with 9 (42.9%) and no hospitals falling above and below the 3SD control limit.

Before the pandemic, 52.4% (11/21) of specialist paediatric surgical centres and 33.0% (31/94) of hospitals without these specialist paediatric surgical centres treated significantly higher proportions (>2 CL above the mean) of CYP with complex appendicitis compared with the rest of the hospitals. There was a small shift during the pandemic period, with a slight increase in the proportion of specialist trusts (57.1%, (12/21)) but fewer non-specialist trusts (28.7% (27/94)) caring for a higher proportion of CYP with complex appendicitis.

Sensitivity

Figure S.D: Main and sensitivity monthly appendicitis admissions, by 0-4s, during the pandemic and the mean monthly admission trends for the previous five years

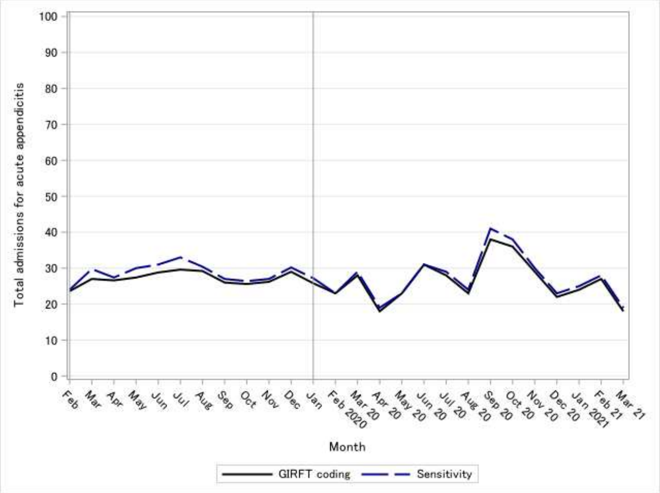

Figure S.E: Main and sensitivity monthly appendicitis admissions, by 5-9s, during the pandemic and the mean monthly admission trends for the previous five years

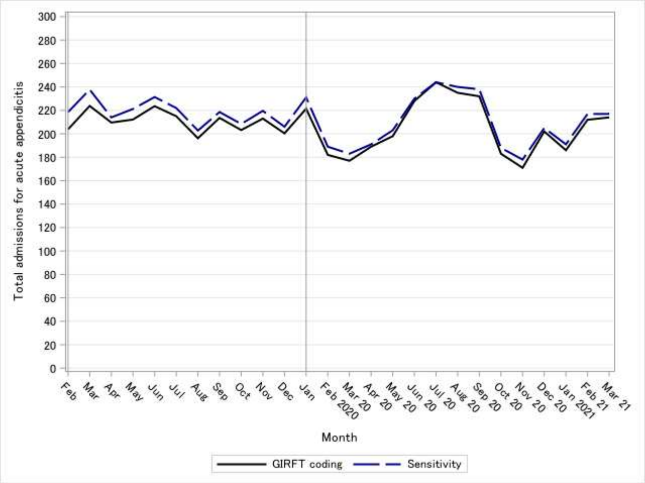

Figure S.F: Main and sensitivity monthly appendicitis admissions, by 10-24 year old's, during the pandemic and the mean monthly admission trends for the previous five years

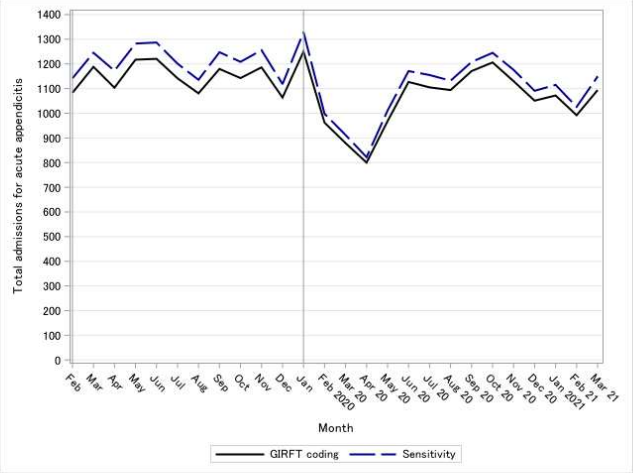

Supplement: Supplementary data [file archdischild-2023-326313supp001.pdf]
